# Evolution of microbiological analytical methods for dairy industry needs

**DOI:** 10.3389/fmicb.2014.00016

**Published:** 2014-02-07

**Authors:** Danièle Sohier, Sonia Pavan, Armelle Riou, Jérôme Combrisson, Florence Postollec

**Affiliations:** ^1^Food Safety and Quality Unit, ADRIA Développement, Agri-Food Technical Institute, Quimper, France; ^2^Bretagne Biotechnologie Alimentaire dairy association member, Analytical Sciences, Danone Research, Palaiseau, France

**Keywords:** dairy, food microbiology, probiotic, analytical method, bacterial fitness, PCR, flow cytometry, quality control

## Abstract

Traditionally, culture-based methods have been used to enumerate microbial populations in dairy products. Recent developments in molecular methods now enable faster and more sensitive analyses than classical microbiology procedures. These molecular tools allow a detailed characterization of cell physiological states and bacterial fitness and thus, offer new perspectives to integration of microbial physiology monitoring to improve industrial processes. This review summarizes the methods described to enumerate and characterize physiological states of technological microbiota in dairy products, and discusses the current deficiencies in relation to the industry’s needs. Recent studies show that Polymerase chain reaction-based methods can successfully be applied to quantify fermenting microbes and probiotics in dairy products. Flow cytometry and omics technologies also show interesting analytical potentialities. However, they still suffer from a lack of validation and standardization for quality control analyses, as reflected by the absence of performance studies and official international standards.

## INTRODUCTION

Fermenting microorganisms play a pivotal role in the development of physicochemical and sensory properties of food products. They also contribute to product safety by limiting the growth of pathogenic and spoilage microorganisms (Caplice and Fitzgerald, 1999). Therefore, evaluation of cell viability is of great importance for the fermented food industry in general, and more specifically for the dairy sector. As fermenting microbes are responsible for organoleptic properties, it is essential to be able to characterize not only cells able to divide but also metabolic activities and bacterial fitness, in order to improve quality controls and end products. For probiotic products, it is important to ensure the presence of sufficient numbers of viable cells that will bring about beneficial health effects (Karimi et al., 2012).

**FIGURE 1:**
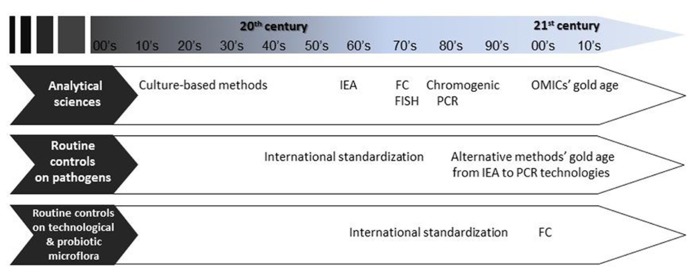
**Evolution of analytical methods, and the gaps between routine controls depending on the targeted microbiota.** IEA, immuno-enzymatic assay; FC, flow cytometry; FISH, fluorescent *in situ* hybridization; PCR, polymerase chain reaction.

The number of food products that involve microbial activities during at least one step of their production is substantial. As a consequence, numerous methods have been described to characterize microbial populations participating in fermentation processes. Traditionally, descriptive culture methods have been used, and remain the most employed to determine the presence/absence of colonies (i.e., cultivable cells) and their numbers. However, these straightforward methods provide a very simplistic, often biased, view of the physiological state of microbial populations in which several subpopulations characterized by various levels of “viability” and metabolic activity may coexist (Davey, 2011). The emergence of molecular techniques has opened new opportunities to characterize the numerous intermediate states of microbial cells, so much so that the well-being, fitness, and metabolic activities are now being targeted through the quantification of biomarkers, rather than just growth/no growth quantifications (Sieuwerts et al., 2008; de Vos, 2011). A biomarker is defined by the National Institutes of Health as “a characteristic that is objectively measured and evaluated as an indicator of normal biological processes, pathogenic processes, or pharmacologic responses to a therapeutic intervention” (Atkinson et al., 2001). We propose an adaptation of this definition to food processes as “a characteristic that is objectively measured and evaluated as an indicator of normal biological processes, pathogenic processes, or cellular responses to food processes.” Molecular methods encompass fluorescent *in situ* hybridization (FISH), flow cytometry (FC), “omics,” and Polymerase chain reaction (PCR)-based technologies. Some of these, such as FC, have been described for a few decades and were successfully used for very diverse research purposes (Díaz et al., 2010), but their routine application to dairy industry analyses is just being seriously considered (**Figure 1**). More recently developed, omics technologies are very promising to better understand microbial communities and to identify biomarkers, but until now they have not been applied to quality control purposes. PCR-based techniques are now being routinely used for the analysis of pathogens and the characterization of technological microbiota in fermented products (Postollec et al., 2011).

What about their standard application to quantify fitness biomarkers? What is the gap between all the described methods and the specific needs of the dairy industry for fast, efficient, reliable, and standardized methods? This article will review the existing methods described to enumerate technological microbiota and characterize their physiological status that contribute to quality control of processes and end-products.

In addition to global Internet search, a total of four international databases were screened for journal articles, books, patents, conferences, and symposia proceedings in the field of food science, food industry, life science, and biomedical information (FSTA®, BIOSIS® Preview, Medline®, Foodline®). As this article aims at providing basis for discussion about the real use of these methods by dairy industry laboratories, current lacks, and future possibilities to facilitate the transfer from research knowledge to food industry applications, only references published between 2000 and 2013 were considered. Only representative publications for each technique and type of application were retained, and priority was given to reviews discussing about specific techniques and their applications. Methods for strain identification or characterization were excluded.

## CULTURE METHODS, OUTDATED OR ESSENTIAL?

The growth of colonies on nutrient agar is routinely used in all microbiology laboratories and is the simplest way to detect and quantify viable microbes. Protocols based on culture methods and colony counting are validated as reference or alternative methods according to the European and International Standard Organization (EN ISO) standards, to detect and enumerate food-borne pathogens, total microbiota and hygiene indicator microbes. A few ISO methods are also available to quantify probiotics and fermenting microbes employed in the dairy industry (Boyer and Combrisson, 2013).

The main limitations of these enumeration methods are the lack of discrimination between the targeted microbes and the endogenous microbiota, the time-to-result, false positive counts, matrix-dependent efficiency, and last but not least, the impossibility to recover viable but non-cultivable (VBNC) cells, which are seen as dead. The concept of viability of microorganisms was for long considered as the ability to multiply on an optimal medium (Postgate et al., 1961). Then, in the 80s, the concept of VBNC was developed (Roszak et al., 1984; Roszak and Colwell, 1987), with the application of additional methods such as Live/Dead detection staining kits or reverse transcription (RT)-PCR, to differentiate cells able to divide, VBNC and dead ones. It is now universally recognized that many intermediate states exist between live and dead bacteria (Kell et al., 1998; Nebe-von-Caron et al., 2000; Díaz et al., 2010) and that colonies recovered by application of a culture method correspond to the cells (or groups of cells) that are able to replicate under the provided growth conditions.

In spite of the evolution of the viability concept, of the increased knowledge relating to the physiological states of microbial cells and of the development of molecular methods that do not display some of the above mentioned limitations, there is still a great interest in culture methods as reflected by the regularly published studies. Various agar plate media were described for selective enumeration of probiotic and non-probiotic lactic acid bacteria (LAB) and *Bifidobacterium* species in yogurt, cheese, or in pure cultures (Tharmaraj and Shah, 2003; Van de Casteele et al., 2006; De Carvalho Lima et al., 2009; Ashraf and Shah, 2011; Saccaro et al., 2011; Karimi et al., 2012). Probiotic survival in commercial products including powders, frozen material, micro-encapsulated cultures, capsules, foods, and drinks was evaluated using various agar plate media (Corcoran et al., 2004; Phillips et al., 2006; Antunes et al., 2007; Magariños et al., 2007; Champagne et al., 2010, 2011; do Espírito Santo et al., 2011; Forssten et al., 2011; Oberg et al., 2011). Probiotic shelf-life during which the minimal content in viable cells has to be guaranteed was determined. The parameters influencing probiotic survival in yogurt and cheese were also studied (Donkor et al., 2007; Karimi et al., 2011). To improve the efficiency of LAB selective media, many variations of the basic agar formulas and culture conditions were proposed, including the use of antibiotics, different incubation temperatures, NaCl concentrations, pH or carbon sources (Van de Casteele et al., 2006; Antunes et al., 2007; Saccaro et al., 2011). However, the limitations already present with older media remain. For microbiologically complex products, the autochthonous microbiota is often undistinguishable from the technological microbiota (Phillips et al., 2006; Ashraf and Shah, 2011). In addition, most culture methods allow discriminating bacteria at the genus level only, and at best at the species level (Tharmaraj and Shah, 2003; Ashraf and Shah, 2011; Saccaro et al., 2011). Matrix effect is another important aspect to consider when applying a culture method. Indeed, protocols developed to enumerate microbes in a given food product may not be reliable with another food product, as exemplified in many studies where variable bacterial counts were found depending on the matrix used (Van de Casteele et al., 2006; do Espírito Santo et al., 2011; Forssten et al., 2011). This underlines the need to develop specific methods, but also the lack of quality controls. Indeed, a majority of the developed methods focus on feasibility aspects but do not demonstrate to what extent the method is accurate, reliable, biased, and do not establish the detection limits. Similarly to the matrix effect, a strain effect may also occur (Van de Casteele et al., 2006), and again, the lack of quality controls to evaluate whether a given method can be accurately extrapolated to other strains is blatant. Considering the currently available cultural tools, it is generally recommended to use an additional identification method (preferably molecular), to ensure the accuracy and reliability of the results. This is especially important with probiotic bacteria enumeration, because minimal viable numbers have to be present in the product to guarantee health effects. Therefore, the need for clear recommendations is obvious. The set-up and validation of methods according to official standards would highly benefit the dairy industry. Recommendations for each analytical step of the viability assessment of probiotics in food products, beverages, dry or frozen preparations have been recently proposed (Champagne et al., 2011). Although specific for probiotics, most of these recommendations can be applied to the analysis of fermented products.

Strikingly, the use of chromogenic media has been developed for the analysis of pathogenic and spoilage bacteria and validated and normalized methods are available, but nothing of the kind was set up for technological microbiota. Indeed, colonies of *Listeria*, *Salmonella*, *Bacillus cereus*, *Escherichia coli,* and other enterobacteria can now be easily distinguished by a large range of colors. Chromogenic media are user-friendly culture methods, and can be used without knowledge in molecular biology. Such media would undoubtedly be very useful for microbiological controls in the dairy industry, and their development would contribute to fill in the lack of specific methods.

## MICROSCOPY, FLOW CYTOMETRY, AND OTHER FLUORESCENT LABELING-BASED METHODS: APPLICABILITY FOR THE FOOD INDUSTRY?

Fluorescent *in situ* hybridization was a popular technique in research laboratories in the 90s. It is based on the identification of cells containing specific nucleic acid sequences. Oligomer probes conjugated to fluorescent molecules hybridize to their target DNA or RNA, and fluorescence is detected by microscopic observation (Bottari et al., 2006). In the last years, a few publications have described the use of FISH to enumerate dairy microbes. Propionibacteria were detected and enumerated in cheese with good correlations with bacterial counts (Babot et al., 2011). García-Hernández et al. (2012) have combined a direct viable count method with FISH to enumerate viable yogurt strains inoculated in feces. FISH allows highly specific bacterial detection and, unlike other molecular techniques, provides information about spacial distribution of bacteria in their environment due to the use of imaging. In spite of these advantages, FISH remains difficult to set up, has low repeatability and artifacts and interferences with the food matrix often occur. Therefore its application for routine analyses is difficult to implement. The use of other fluorescent labeling techniques, sometimes combined with FISH, is also described (Moreno et al., 2006; Zotta et al., 2012). Fluorescent labeling is always associated with microscopy technologies. While the performances of methods based on fluorescent labeling and microscopic detection are usually very good (Auty et al., 2001; Bernardeau et al., 2001), a routine use of epifluorescence and/or confocal microscopy for food industry quality controls is hardly conceivable.

Another technology that has greatly evolved together with the development of fluorescent dyes is FC. It allows characterizing individual cells within a cell population, and to discriminate between various VBNC and physiological states (Sheehan et al., 2005; Lahtinen et al., 2006; Papadimitriou et al., 2006, 2007; Quiros et al., 2007; Sunny-Roberts and Knorr, 2008; El Arbi et al., 2011; Zotta et al., 2012), which is a major advantage over culture-based methods. The excellent review from Díaz et al. (2010) describes the principle of FC, a range of fluorescent probes associated with various cellular functions and applications to industrial microbial bioprocesses including dairy industry applications. Nowadays, FC appears as the most promising labeling technology (Tracy et al., 2010; Davey, 2011) and many publications describe its use in food microbiology. A large choice of dyes is available to target cell components (nucleic acids, proteins, lipids), intracellular pH, membrane integrity, intracellular ions, viability markers, and membrane energization (Maecker et al., 2004; Sheehan et al., 2005; Papadimitriou et al., 2006; Berney et al., 2007; Quiros et al., 2007; Comas-Riu and Rius, 2009; Díaz et al., 2010; Doherty et al., 2010; El Arbi et al., 2011; Zotta et al., 2012). A fine characterization of the cellular status is possible by combining several dyes (Chen et al., 2011). The effects of acid, oxidative, osmotic, or cold stresses encountered during food production or storage on the cellular status were studied (Ananta et al., 2005; Lahtinen et al., 2006; Moreno et al., 2006; Papadimitriou et al., 2007; Sunny-Roberts and Knorr, 2008; Kramer et al., 2009; El Arbi et al., 2011; Zotta et al., 2012). Population dynamics in batch cultures can also be characterized (Quiros et al., 2007; Comas-Riu and Rius, 2009; Lopes Da Silva et al., 2009), as well as early detection of bacteriophage infection (Michelsen et al., 2007), membrane changes at various cheese cooking temperatures (Sheehan et al., 2005), and antibacterial effects of bacteriocins (Budde and Rasch, 2001). FC has also been proposed as a means to enumerate viable probiotic populations in commercial products (Maukonen et al., 2006). Correlations between FC enumerations and plate counts are often very good during exponential growth and when cells are not submitted to stress (Bunthof and Abee, 2002; El Arbi et al., 2011). In other situations FC counts generally outnumber the plate counts by 0.3–1 log of CFU/g (Gunasekera et al., 2000; Quiros et al., 2007; Sunny-Roberts and Knorr, 2008). Among the other advantages of FC over traditional culture methods, one should mention the shorter analysis time and possible automation (Maukonen et al., 2006; Díaz et al., 2010; Davey, 2011). However, while a whole population cellular activity can be characterized, distinction between near genera or species remains difficult because specific nucleic acid and antigen probes have not yet been extensively used. In addition, the quantification – and sometimes detection – limit is relatively high, for instance 10^3^–10^4^ cells/ml in milk (Gunasekera et al., 2000; Maecker et al., 2004). Protocol development and data analyses also require some experience (Davey, 2011). Indeed, depending on their composition some samples need additional preparation, such as a reduction of protein amounts (Gunasekera et al., 2000; Doherty et al., 2010). False-positives or data dispersion may be observed with some dyes (Maecker et al., 2004).

In spite of the few limitations, FC appears as a very promising tool for the food industry (Díaz et al., 2010; Tracy et al., 2010; Davey, 2011). Beyond the simple enumeration of cells, FC can provide higher knowledge about microbial fitness and metabolic activities during bioprocesses. Therefore, this will improve optimization of technological processes involving dairy bacteria, prediction of microbial performances along the whole process and the presence/absence of activity during storage. Such application would, for instance, benefit the quality control of probiotic products during their shelf life, and inclusion of FC as a validated technology for this purpose is supported by some authors (Lahtinen et al., 2006). Today, existing kits and automated systems are rather dedicated to the detection of industrial and environmental contaminants (Díaz et al., 2010). But FC is currently used for milk quality control, and an ISO standardized method is being set-up under coordination of the International Dairy Federation, for enumeration of LAB in starter cultures and their applications (International Dairy Federation, 2012). An automated system is being commercialized by AES-Chemunex to enumerate viable bacteria in industrial products (). An important step is still to overcome for FC routine analysis, and developed applications require thorough calibration and validation of their performances and limitations.

## OMIC METHODS: RESEARCH TOOLS ONLY?

The number of sequenced lactobacilli and lactococci genomes has greatly increased during the last decade (Liu et al., 2005; Makarova et al., 2006; de Vos, 2011). High throughput functional genomics and comparative metagenomic studies have also known a strong expansion and many recent articles describe research applications of omics technologies in the field of food fermentations and associated microbes. Transcriptomics and proteomics studies have enabled analyzing the response of LAB to various conditions of culture, stresses and industrial processes (de Vos, 2011). For instance, several articles reported the use of omics to characterize gene expression and protein synthesis during growth in milk or cheese and bacterial adaptation during commercial preparation (Gitton et al., 2005; Raynaud et al., 2005; Herve-Jimenez et al., 2008; Azcarate-Peril et al., 2009; Cretenet et al., 2011; Taibi et al., 2011; Thevenard et al., 2011). Transcriptomic, proteomic, and metabolic responses of LAB and bifidobacteria to temperature, acidic or oxidative conditions, bile, osmotic stress, limited carbon sources, or basic pH were described (De Dea Lindner et al., 2007; Cretenet et al., 2011; Lee et al., 2011; Li et al., 2011). The behavior of LAB in mixed cultures in milk, yogurt, cheese or probiotic products was also evaluated (Herve-Jimenez et al., 2008; Sieuwerts et al., 2008, 2010; Ruiz et al., 2009; Thevenard et al., 2011). Moreover, Omics technologies were applied to study genome evolution and biodiversity in various ecological niches (Siezen et al., 2004, 2008; Makarova et al., 2006; Rasmussen et al., 2008; O’Sullivan et al., 2009; Pastink et al., 2009; Taibi et al., 2011). One of the goals of these omics studies is to identify key biomarkers that could be used for the screening of new probiotic or technologically interesting strains and for the evaluation of their physiological states in order to improve functionality during industrial processes (Reid et al., 2002; Ventura et al., 2007; Rasmussen et al., 2008; Izquierdo et al., 2009; Sieuwerts et al., 2010; Thierry et al., 2011). The wealth of data generated by omics approaches also necessitates powerful tools to analyze and interpret them. It is interesting to mention the method developed by Segata et al. (2011) available as an online interface, to quickly select for candidate biomarkers by screening metagenomic data.

Usefulness of omics technologies to understand microbial behaviors and cellular pathways with the aim to optimize industrial processes is obvious. By contrast, applications of these high throughput tools to improve analytical methods have been neglected. However, identification of biomarkers that could help developing new simple and fast analytical methods for quality controls deserves attention.

## THE POPULAR PCR AND PCR-BASED METHODS: READY FOR ROUTINE ANALYSES?

Since its development in the 80s, PCR has become fundamental to biological and medical research laboratories (Bartlett and Stirling, 2003). Widely employed for descriptive purposes such as the detection of microbes and analyses of ecosystems composition in combination with other technologies, it is now routinely used for the detection of pathogenic and spoilage microbes in food products (Postollec et al., 2011). Detection of food-borne pathogens by PCR is recognized by the ISO and standardized through several guidelines (ISO, 2005a,b, 2006a,b, 2011a,b). PCR is also used to confirm characteristic colonies from agar plates, as specified by ISO (ISO, 2007). In the last decade, this amplification technique has strongly evolved toward quantitative PCR (qPCR; Masco et al., 2007; Malorny et al., 2008; Le Dréan et al., 2010), and ISO guidelines describing the use of qPCR for the detection of food-borne pathogens in foodstuff have also been developed (ISO, 2012, 2013). The last 10 years have witnessed a large number of research articles and reviews describing PCR-based applications for probiotics and microbes involved in fermentation processes. Arisen from comparative genomics analyses, these methods aim at specifically quantifying microbial populations (Friedrich and Lenke, 2006; Masco et al., 2007; Randazzo et al., 2009; Sheu et al., 2009, 2010; Abdulamir et al., 2010; Reimann et al., 2010; Sohier et al., 2012), at assessing the role of microbes in dairy products processes (Collado et al., 2006; Ben Amor et al., 2007; Randazzo et al., 2009; Masoud et al., 2011), or at allowing simultaneous identification of dairy and probiotic bacteria containing multiple strains (Wong and Medrano, 2005; Sul et al., 2007; Senan et al., 2008; Smith and Osborn, 2009).

Polymerase chain reaction techniques were also used to evaluate physiological states and viability of microorganisms during products processes (Lahtinen et al., 2008; Marco and Kleerebezem, 2008; García-Cayuela et al., 2009; Randazzo et al., 2009; Matijašić et al., 2010; Meng et al., 2010; Bove et al., 2011). One way to distinguish between viable and dead bacteria is to use RT-qPCR which targets RNA instead of DNA (Matsuda et al., 2009; Falentin et al., 2010; Reimann et al., 2010). However, due to the short and variable half-life of RNA molecules and difficulties to extract high quality RNA from complex matrices, the reliable use of RT-qPCR remains delicate and necessitates thorough quality controls and standardizations (Postollec et al., 2011). Other alternatives have been proposed, among which is the viability PCR approach (Fittipaldi et al., 2012). DNA intercalating agents such as propidium monoazide (PMA) and ethidium monoazide (EMA) are used. They penetrate only into dead cells with compromised membranes and subsequently prevent amplification of DNA by PCR. Quantification of viable cells in probiotic products or viable LAB in fermented milk was proposed using EMA-PCR and PMA-PCR, and showed good correlations with plate counts (García-Cayuela et al., 2009; Matijašić et al., 2010; Meng et al., 2010). RT-qPCR is a method of choice to study physiological states. However, in spite of the increasing availability of omics data, and due to the above mentioned difficulties to work with RNA molecules, validated methods to quantify bacterial fitness biomarkers still lag behind.

Compared to standard plate counts, the reduced time-to-results and higher specificity of PCR-based methods make them very appropriate for dairy industry needs (Boyer and Combrisson, 2013). However, a big step leading to routine use for dairy quality controls is still present because many studies do not take into account these specific needs. Indeed, robustness, accuracy and limits of the developed methods are rarely characterized, and validations on final products are not always performed.

A drawback of the high sensitivity of PCR and RT-qPCR is that these methods are also very sensitive to small variations in sample preparation, amplification and mode of data expression, which may have a major impact on the results. The lack of consensus on how best to perform experiments and interpret PCR data is regularly pointed out (Bustin, 2009; Boyer and Combrisson, 2013). MIQE guidelines were published in order to ensure reproducibility and comparability of data (Bustin et al., 2009). Recently, a dMIQE checklist proposing the minimum information for publication of digital PCR (dPCR; see below) requirements was set up (Huggett et al., 2013). Based on the current lack and knowledge we previously proposed a list of recommendations for better use of qPCR in food industry analyses (Postollec et al., 2011). An ISO standard defines the minimal requirements for the detection of food-borne pathogens in foodstuffs by qPCR (ISO, 2011b) but no similar standard is available for fermenting or probiotic microbes. All these recommendations should help designing qPCR-based methods as alternative methods for quantification of dairy microorganisms that would be further validated according to EN ISO 16140 (ISO, 2003a).

Digital PCR or droplet digital PCR (ddPCR) that uses the well-known principle of the most probable number (MPN) employed in microbiology appears as an interesting alternative to qPCR (Baker, 2012). It is based on amplification of single target DNA molecules, and thus provides absolute quantification without the need to set up a standard curve, which would be more accurate than qPCR and should facilitate comparability of data (White et al., 2009). As for standard qPCR, dPCR can also be multiplexed, thus reducing the bias coming from the competition between targets (Ottesen et al., 2006; Hua et al., 2010; Zhong et al., 2011). Applications of dPCR for the detection or quantification of microbes in food have not yet been described. Clinical microbiology studies have reported good performances of the method and good correlations with qPCR (Hayden et al., 2013). Other interesting clinical and environmental applications [for instance, Devonshire et al., 2013; Heredia et al., 2013; Kelley et al., 2013; Song et al., 2013] suggest a promising future for dPCR that could easily be extended to food microbiology.

## EXISTING PATENTS AND STANDARDS

Very few patents targeting nucleic acid sequences of LAB were deposited. They relate to nucleic acid sequences of *L. acidophilus* encoding cell-surface protein homologues and stress-related proteins and their uses (Klaenhammer et al., 2004, 2005), *L. rhamnosus* polynucleotides, probes, primers, genetic constructs, polypeptides, and methods for using them (Dekker et al., 2002, 2004; Glenn et al., 2004), *Lactobacillus* nucleic acids and proteins involved in stress response and recombinant expression vectors and host cells (Klaenhammer et al., 2006, 2010). A patent describing a method to detect live microbiological contaminants in food, by detecting a mRNA coding for an elongation factor synthesized by the contaminants, is also available (Gendre and Brignon, 1998). The nucleic acid molecules and metabolic products described in these patents could be used as biomarkers to characterize bacterial activity and fitness.

Only a few ISO standards relating to fermenting LAB or bifidobacteria in dairy products are available. Some of these standards specify plate-count methods for the enumeration of targeted microbiota in milk or yogurt (ISO, 1998, 2003c, 2006c, 2010b). Some others do not refer to bacterial counts but specify the tests for the identification of the characteristic microorganisms in yogurt or the composition of starters (ISO, 2003b, 2010a).

## CONCLUSION

**Table 1** summarizes the current uses and lacks of the main analytical methods in dairy microbiology. Indeed, many databases are now available and provide a deeper understanding of the physiology and metabolic characteristics of strains. Still underexploited, they could help developing routine methods to evaluate LAB and probiotics physiological states. For instance, the current biological and physiological knowledge about fermenting bacteria should help developing chromogenic media based on biochemical reactions specific for intrinsic strain properties. These chromogenic tools are very straightforward, more specific than classical agar media, and very relevant for use in microbiological controls performed by the dairy industry.

**Table 1:** Current uses and lacks of the most employed analytical methods.

Method	Use	Requirements for deployment in the dairy industry
Culture-based methods	Selective enumeration of LAB and bifidobacteria in pure cultures, yogurt, cheese	Higher selectivity Determination of the accuracy, limits and reliability
	Survival of probiotics in commercial preparations	Validation as alternative methods according to ISO standards
	Determination of shelf life of probiotic products that ensure sufficient viable cell numbers	
	Study of parameters influencing probiotic survival	
PCR-based methods	Quantification of specific populations	Benchmark analytical methods
	Study of the role of bacteria in processes	Validated studies for the quantification of biomarkers
	Multiplex identifications	Determination of the accuracy, limits, and reliability
	Study of physiological state and viability during processing	Validation as alternative methods according to ISO standards
	Differentiation between viable and non-viable	
Flow cytometry	Characterization of cellular states in stress conditions (acidic, oxidative, osmotic, cold stress)	Determination of the accuracy, limits, and reliability Validation as alternative methods according to ISO standards
	Population dynamics in batch cultures	
	Study of bacterial fitness to help optimizing processes	
	Early detection of bacteriophages	
	Membrane changes during cheese cooking	
	Antimicrobial effects of bacteriocins	
	Enumeration of viable probiotics in commercial preparations	

Promising FC methods were described for analyses of mixed cultures in milk, yogurt, and other fermented milk products. They could be applied for quality controls during processes or during product shelf life. Nevertheless, they would require careful optimization to prevent artifacts due to the presence of milk proteins. One must also keep in mind that the quantification limits remain high and that current probes are not specific for a species or genus but for a target cell component. Therefore FC would rather be used to characterize physiological states than for specific counts.

OMICS data have allowed identifying many key biomarkers, which could be quantified in order to quickly characterize physiological states. PCR is already routinely used to detect food-borne pathogens and spoilage microbes. This could easily be extended to fermenting and probiotic bacteria, providing standardization of nucleic acid extraction, PCR procedures, and data interpretation. PCR (and PCR-based methods) would be a useful tool to quickly select interesting fermenting strains, or to ensure that optimal conditions are present during processes. Today, standards, patents, or commercial kits that would allow to quickly transferring methods to study fitness and physiological states of LAB and probiotics are not available.

Common to all the techniques reviewed in this study, two major gaps were identified: evaluation of the methods in agreement with the specific needs of the dairy industry (characterization of the robustness, accuracy, and limits) and validation according to official standards (**Table 1**). Indeed, while standard methods have been developed and validated for food-borne pathogens, LAB and probiotics seem to be the poor relations of the diagnosis industry and method standardization (**Figure 1**). This is surprising when considering the amount of quality controls performed by the dairyindustry.

## AUTHOR CONTRIBUTIONS

Danièle Sohier has designed the study, analyzed the data, and reviewed the manuscript. Sonia Pavan has written the manuscript. Armelle Riou has collected the data. Jérôme Combrisson and Florence Postollec have reviewed the manuscript and participated in the discussion.

## Conflict of Interest Statement

The authors declare that the research was conducted in the absence of any commercial or financial relationships that could be construed as a potential conflict of interest.
